# Identification of molecular pathways affected by pterostilbene, a natural dimethylether analog of resveratrol

**DOI:** 10.1186/1755-8794-1-7

**Published:** 2008-03-20

**Authors:** Zhiqiang Pan, Ameeta K Agarwal, Tao Xu, Qin Feng, Scott R Baerson, Stephen O Duke, Agnes M Rimando

**Affiliations:** 1 United States Department of Agriculture, Agricultural Research Service, Natural Products Utilization Research Unit, University, Mississippi 38677, USA; 2 National Center for Natural Products Research, School of Pharmacy, University of Mississippi, University, Mississippi, 38677, USA

## Abstract

**Background:**

Pterostilbene, a naturally occurring phenolic compound produced by agronomically important plant genera such as *Vitis *and *Vacciunium*, is a phytoalexin exhibiting potent antifungal activity. Additionally, recent studies have demonstrated several important pharmacological properties associated with pterostilbene. Despite this, a systematic study of the effects of pterostilbene on eukaryotic cells at the molecular level has not been previously reported. Thus, the aim of the present study was to identify the cellular pathways affected by pterostilbene by performing transcript profiling studies, employing the model yeast *Saccharomyces cerevisiae*.

**Methods:**

*S. cerevisiae *strain S288C was exposed to pterostilbene at the IC_50 _concentration (70 μM) for one generation (3 h). Transcript profiling experiments were performed on three biological replicate samples using the Affymetrix GeneChip Yeast Genome S98 Array. The data were analyzed using the statistical methods available in the GeneSifter microarray data analysis system. To validate the results, eleven differentially expressed genes were further examined by quantitative real-time RT-PCR, and *S. cerevisiae *mutant strains with deletions in these genes were analyzed for altered sensitivity to pterostilbene.

**Results:**

Transcript profiling studies revealed that pterostilbene exposure significantly down-regulated the expression of genes involved in methionine metabolism, while the expression of genes involved in mitochondrial functions, drug detoxification, and transcription factor activity were significantly up-regulated. Additional analyses revealed that a large number of genes involved in lipid metabolism were also affected by pterostilbene treatment.

**Conclusion:**

Using transcript profiling, we have identified the cellular pathways targeted by pterostilbene, an analog of resveratrol. The observed response in lipid metabolism genes is consistent with its known hypolipidemic properties, and the induction of mitochondrial genes is consistent with its demonstrated role in apoptosis in human cancer cell lines. Furthermore, our data show that pterostilbene has a significant effect on methionine metabolism, a previously unreported effect for this compound.

## Background

Pterostilbene is a naturally-occurring phytoalexin identified in several plant species. It belongs to a group of phenolic compounds known as stilbenes, and is found in the heartwood of sandalwood (*Pterocarpus santalinus*) [1] and *P. marsupium *[2]. It was also identified in the leaves of *Vitis vinifera *[3], in infected grape berries of var. Chardonnay and Gamay [4], and in healthy and immature berries of var. Pinot Noir and Gamay [5]. Pterostilbene has also been found in berries of some *Vacciunium *species [5]. Pterostilbene, one of the most extensively studied secondary metabolites found in grapes and wine, is a dimethylether analog of resveratrol that is well known for its hypolipidemic activity. A considerable amount of research effort has been expended to address the biochemical and physiological effects of pterostilbene in animal and microbial systems. For example, the antioxidative activity of pterostilbene was first demonstrated *in vitro *by its inhibition of methyl linoleate oxidation [6]. Pterostilbene was reported to scavenge 1,1-diphenyl-2-picryl-hydrazyl (DPPH) free radicals and to inhibit the oxidation of citronellal, and lipid peroxidation in rat liver microsomes and in cultured human fibroblasts [7]. Pterostilbene isolated from *Anogeissus acuminata *(Family Combretaceae) is cytotoxic against a number of cancer cell lines, including human breast cancer and murine lymphoid neoplasma cells [8,9]. More recently, it has been demonstrated that pterostilbene can reduce cholesterol levels *in vivo *in hamsters through the activation of the peroxisome proliferator-activated receptor α (PPARα) [10]. Pterostilbene has been reported to reduce glucose and increase plasma insulin levels significantly in normal and diabetic rats [11]. Furthermore, pterostilbene has been shown to be cancer-chemopreventive [8,12] and anti-inflammatory [13].

Investigation of the pathogen-host interactions of *Vitis vinifera *has led to the hypothesis that resistance is not due to preformed physical or chemical factors, but rather to an active defense mechanism that is triggered by the pathogen, of which stress metabolites including resveratrol, α-viniferin and ε-viniferin are an important component [14]. Pterostilbene, produced in leaf tissues by various species of the *Vitaceae *family following fungal infection, proved to have more potent antifungal activity than resveratrol (reviewed in [3,15,16]). However, the mechanism by which pterostilbene inhibits fungi is not well understood. Results from early studies suggested that the biological activities of the compound mainly involved effects on the plasma membrane [5,17], and destruction of ribosomes, endoplasmic reticulum, and mitochondrial membranes [17]. Further information on its precise mechanism of action would be useful not only for its potential development as a drug, but also in understanding its ecological significance to producing plant species. In the present study, using transcript profiling analysis, we monitored the gene expression profile of yeast cells treated with pterostilbene in an effort to identify the molecular pathways affected by this compound.

## Methods

### Yeast strains and media

*S. cerevisiae *S288C (*MATα, SUC2, mal, mel, gal2, CUP1, flo1, flo8-1*), obtained from ATCC (Manassas, VA), was used in the microarray experiments. The deletion strains and the isogenic wild type strain (BY4742) were obtained from Open Biosystems (Huntsville, AL). Synthetic dextrose (SD) medium, containing 0.67% (w/v) yeast nitrogen base without amino acids and 2% (w/v) dextrose, was used to grow the wild type S288C strain. Standard Yeast Peptone Dextrose (YPD) medium (1% yeast extract, 2% peptone, 2% dextrose) was used to grow the deletion strains and their respective isogenic wild type control. In all cases, the medium was buffered with 0.165 M 3- [N-Morpholino]propanesulfonic acid (MOPS) (Sigma-Aldrich Corp.) and the pH was adjusted to 7.0 with NaOH.

### IC_50 _determination and yeast cultures for microarray studies

IC_50 _value (concentration of inhibitor required for 50 percent inhibition of growth) was first determined in a conventional microplate assay. The obtained IC_50 _was then further confirmed in 50-ml large-scale cultures as previously described in order to closely mimic the microarray experimental conditions [18]. For microarray experiments, a single colony of *S. cerevisiae *was inoculated into 25 ml of SD medium and grown overnight at 30°C with shaking at 200 rpm. The culture was used to inoculate 50-ml of SD medium to an A_600 _of 0.1. Three independent 50-ml cultures were grown for pterostilbene treatments. In parallel, another set of three independent cultures were grown for solvent treatments which served as the untreated controls. When the cultures reached an A_600 _of 0.2, pterostilbene was added at 70 μM concentration from a stock solution of 0.5 M dissolved in dimethyl sulfoxide (DMSO). In the control cultures, 0.25% of DMSO was simultaneously added. The cultures were allowed to grow until an A_600 _of 0.5 was reached [18]. Cells were harvested by centrifugation at 500 × *g *in a Sorvall centrifuge using a SH-3000 rotor. The cells were flash-frozen in liquid nitrogen and stored at -80°C until use.

### RNA preparation and hybridization to Affymetrix GeneChips

Total RNA was isolated using the Qiagen RNeasy^® ^Midi-kit (Qiagen, Inc., Valencia, CA) with modifications as previously described [18]. RNA was processed for target preparation using the GeneChip One-Cycle Target Labeling and Control Reagents kit (Affymetrix, Santa Clara, CA), according to the manufacturer's protocol. Microarray hybridization was performed using the Affymetrix GeneChip Yeast Genome S98 Array using protocols described by Affymetrix (Santa Clara, CA). All microarray data are deposited at the Gene Expression Omnibus as GSE10554.

### Microarray data analysis

Normalized data from the Affymetrix software were analyzed using the GeneSifter microarray data analysis system (VizX Labs LLC, Seattle, WA; [19]). This program identifies differentially expressed genes and establishes the biological significance based on Gene Ontology (GO) classification [20,21] into biological process, molecular function and cellular component. The GeneSifter program also produces *z*-score reports. A *z*-score is a statistical rating of gene ontologies and indicates whether each GO term occurs more or less frequently than expected. The differential expression of genes was calculated by averaging the normalized triplicate samples and running a pairwise analysis [22,23]. Statistical significance was determined using Student *t*-test (two tail, unpaired) with correction factor for false discovery rate (Benjamini and Hochberg) included [24]. Gene lists generated using GeneSifter were further analyzed using the *Saccharomyces *Genome Database [25].

### Quantitative real-time RT-PCR assay

To confirm the results from microarray experiments, real-time quantitative RT-PCR was carried out using the same RNA preparations that were used in the microarray experiments. Total RNA was treated with DNase I and the RT-PCR reaction was performed as described [18]. The sequences of the primers for each gene selected for the assays are listed in Table 1. Additionally, linear regression analysis as well as an F-Test to assess goodness-of-fit was performed on log_2_-transformed expression ratios (pterostilbene treated:untreated cells) obtained from real-time RT-PCR and microarray experiments (see Additional file 1: Correlation between expression ratios obtained from quantitative real-time RT-PCR and microarray experiments) using the Microsoft Excel Analysis ToolPak add-in module, and a statistically-significant (*p *< 0.05) correlation between the two methods was indicated.

**Table 1:** Gene-specific primer sequences for quantitative real-time RT-PCR analysis

**Gene name**	**Forward Primer**	**Reverse primer**	**ORF**
*BAG7*	5'-TGCTTCCAAGGTCACGTGC-3'	5'-GATGTATGGTAAAATTGTTGGAGTCAG-3'	YOR134W
*RSB1*	5'-ACCCGTTGTTGTTACCGTTTG-3'	5'-GATAGCCATCCCAACCGACA-3'	YOR049C
*UPC2*	5'-TTCGAGGACTGAAACTGGACTG-3'	5'-TCTAGGCGGTGAGATGAAACG-3'	YDR213W
*OAF1*	5'-CAGTGGTTTACGTCAGGGTCC-3'	5'-CGTTTGCTCTTTTGAACCACC-3'	YAL051W
*INO4*	5'-ATGAGGCAAAAACCGGCAG-3'	5'-TTTGTTCTTGTACGGGATCGC-3'	YOL108C
*MET3*	5'-GTGCCTCCCACTTCATTGTTG-3'	5'-GAGTTCTTACCTGGGCCCG-3'	YJR010W
*AZR1*	5'-TATTACCTGGCGTTGCTTTCG-3'	5'-TGAGAAGCGAGCATGGAACTT-3'	YGR224W
*PDR3*	5'-CTGCGGCATCAAGATCGAT-3'	5'-GCTAGGCGCAGAATGTTGTCT-3'	YBL005W
*RTG1*	5'-CAGTAAGACCGCCAGCAAAGA-3'	5'-CACCATACCCTCCGTACTCGA-3'	YOL067C
*RTG3*	5'-CAATTGGGAGCCACCGTTAT-3'	5'-CCAGCATGATTGTGGTTACCG-3'	YBL103C
*RLM1*	5'-AAATACCGGGCTGACTCCATAC-3'	5'-GTGCCTAGTGGTGTTTGAGCAG-3'	YPL089C

### Sensitivity assays with yeast mutants

Cells were grown overnight in YPD broth (pH 7.0, MOPS buffered) until the optical density of 3.0 at A_600 _was reached. For mutants, G418 at the concentration of 200 μg/ml was added to the broth. Seven serial 1:5 dilutions of the overnight cultures were prepared in appropriate medium and plated on YPD agar containing either 110 μM of pterostilbene or 1% DMSO as control. The plates were photographed after incubating for 2 days at 30°C.

## Results

### IC_50 _determination in *S. cerevisiae*

**Figure 1:**
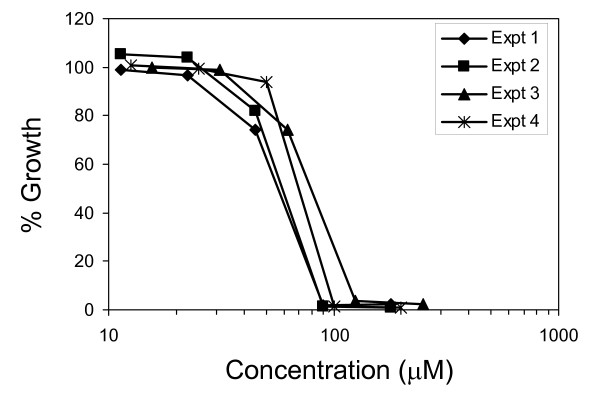
Determination of IC_50 _concentration for pterostilbene in yeast cells. Results are shown from a microtiter assay (experiment 1) and three independent assays performed in large scale cultures (experiment 2–4) as described in "Methods".

Pterostilbene is known to possess strong antifungal activity [16]. In this study, in order to investigate how pterostilbene functions in fungal cells, we determined the consequences of exposure to this compound by monitoring the whole-genome transcriptional response in the model yeast *Saccharomyces cerevisiae*. We made use of *S. cerevisiae *since it has been extensively used for elucidating the molecular targets of antifungal and therapeutic compounds (reviewed in [26,27]). We first determined the IC_50 _value for pterostilbene in *S. cerevisiae *cells using a combination of microplate-based and large-scale culture assays as previously described [18]. The IC_50 _was determined to be 70 μM based on the average value from four independent experiments (Figure 1), and this concentration was used for subsequent microarray studies.

### Categories of genes responding to pterostilbene

Our results demonstrate that pterostilbene significantly (*p *< 0.05) affected the expression of over a thousand genes under our experimental conditions, with the up- and down-regulation of 1007 and 182 genes, respectively (see Additional file 2: List of all significant pterostilbene-responding genes). The most immediate observation is the very high fraction of up-regulated genes, indicating many different biological processes are affected by pterostilbene (Table 2). Of these, 44 genes were up-regulated more than 10-fold. The majority of the genes showed fold-changes ranging between 2.0 to 5.0, while approximately 200 genes showed fold-changes of 5.0 to10.0 (Table 2).

**Table 2:** Distribution of differentially expressed genes based on fold-change of expression

**Threshold**	**Number of differentially expressed genes**	**Number of genes up-regulated**	**Number of genes down-regulated**
≥ 2 to <5-fold	1032	863	169
≥ 5 to <10-fold	113	102	11
≥ 10 to <50-fold	37	35	2
≥ 50-fold to <100-fold	5	5	0
≥ 100-fold	2	2	0

To explore the biological significance of the observed transcriptome response to pterostilbene, a *z*-score report was generated (Table 3) using the GeneSifter software [19]. A positive *z*-score indicates that more genes than expected by chance alone fulfill the criterion in a specific ontology term, while a negative *z*-score indicates that the term occurs less frequently than expected [22]. Pterostilbene affected the expression of genes that were associated with a diverse array of biological, molecular and cellular ontologies. As shown in Table 3, the most significantly enriched ontologies identified for the up-regulated genes included "response to drug," "regulation of transcription from PolII promoter," "mitochondrion organization and biogenesis," and "protein folding," with *z*-up scores of 5.05, 2.4, 2.12, and 4.24 respectively (Table 3, under Biological Process). Related processes were also significantly over-represented under Molecular Function, as represented by the following categories: "ABC transporter activity," "transcription factor activity," "ATPase activity," "peptidase activity," and "chaperone activity," with *z*-scores of 3.02, 4.67, 2.24, 2.12, and 3.56 respectively (Table 3, under Molecular Function). Among the down-regulated genes, terms associated with the biological process "amino acid and derivative metabolism" (specifically, the subcategory of "methionine metabolism"), and the cellular component "cell wall" were over-represented with *z*-scores of 5.08 and 8.22, respectively.

**Table 3:** *Z*-score reports for selected gene ontologies represented in the transcription profile of pterostilbene-treated yeast cells

**Ontology**	**Genes**	**Up**	**Down**	**Array**	***z*-up**	***z*-down**
Biological process						
Biosynthesis	150	104	**46**	871	-2.73	**2.7**
Response to stress	83	**76**	7	365	**3.35**	-1.92
*Response to drug*	*13*	***13***	*0*	*26*	***5.05***	*-1.01*
Amino acid and derivative metabolism	42	22	**20**	189	-1.27	**5.08**
*Sulfur amino acid metabolism*	*14*	*2*	***12***	*30*	*-1.27*	***10.5***
*Aspartate family amino acid metabolism*	*14*	*1*	***13***	*41*	*-2.25*	***9.48***
*Methionine metabolism*	*11*	*0*	***11***	*21*	*-1.92*	***11.78***
Regulation of transcription from Pol II promoter	40	**36**	4	169	**2.4**	-0.96
Reproduction	32	18	**14**	176	-1.77	**3.01**
Ion transport	27	18	**9**	104	0.71	**2.68**
Regulation of cell cycle	24	15	**9**	122	-0.81	**2.15**
Mitochondrion organization and biogenesis	20	**18**	2	77	**2.12**	-0.53
Protein folding	19	**17**	2	46	**4.24**	0.22
Molecular function						
Transferase activity	99	67	**32**	604	-2.76	**2.11**
Transporter activity	87	63	**24**	406	0.49	**2.36**
*Ion transporter activity*	*27*	*18*	***9***	*121*	*0.05*	***2.13***
*Organic acid transporter activity*	*12*	*7*	***5***	*46*	*0.1*	***2.52***
*ATP-binding cassette (ABC) transporter activity*	*10*	***10***	*0*	*29*	***3.02***	*-1.08*
Oxidoreductase activity	59	38	**21**	236	0.63	**4.23**
ATPase activity	33	**33**	0	158	**2.24**	-2.56
Peptidase activity	25	**25**	0	116	**2.12**	-2.18
Chaperone activity	24	**24**	0	85	**3.56**	-1.86
Transcription factor activity	21	**20**	1	54	**4.67**	-0.76
Kinase regulator activity	12	5	**7**	36	-0.14	**4.93**
Cellular component						
Mitochondrion	161	**148**	13	644	**6.1**	-2.23
Plasma membrane	57	38	**19**	215	1.13	**4.31**
Endoplasmic reticulum	52	**37**	15	364	**-2.68**	0.63
Cytosol	38	**26**	12	390	**-4.8**	-0.51
Cell wall	36	18	**18**	95	1.09	**8.22**
Chromosome	22	**14**	8	182	**-2.81**	0.64
Cytoskeleton	22	**16**	6	194	**-2.68**	-0.34

*Z*-scores were determined using GeneSifter software. Criteria for inclusion in the table were an ontology having a *z*-score ≥ 2 or ≤ -2 and containing ≥ 10 genes. Genes, number of genes differentially expressed within the assigned ontology; array, the number of genes on the microarray assigned to the particular gene ontology. Sub-categories within a given ontology are shown in italics. Significant *z*-scores and corresponding numbers of genes in each category are shown in bold.

As mentioned, pterostilbene is involved in fatty acid and lipid catabolism through activation of the peroxisome proliferator-activated receptor α (PPARα) [10]. With respect to the gene ontology analysis (Table 3), the effect on lipid metabolism by pterostilbene was observed to be less statistically significant, however further examination revealed several important genes associated with lipid metabolism within the microarray data (Table 4). These included several genes involved in fatty acid breakdown including *FOX2 *(multifunctional beta-oxidation protein), *ECI1 *(peroxisomal Δ3, Δ2-enoyl-CoA isomerase), and *OAF1 *(oleate-activated transcription factor). In addition, many genes involved in the biosynthesis of fatty acids, phospholipids and sphingolipids were up-regulated by pterostilbene treatment, as well as 8 genes involved in the regulation of lipid metabolism. The latter include *OAF1 *(involved in regulation of beta-oxidation of fatty acids) [28,29], *UPC2 *(involved in regulation of sterol biosynthesis and transport) [30], and *INO4 *(involved in regulation of phospholipid metabolism) [31]. Furthermore, two additional genes, *MGA2 *and *SPT23*, encoding endoplasmic reticulum membrane proteins, Spt23p and Mga2p, respectively, were also up-regulated. Spt23p and Mga2p regulate expression of *OLE1*, a gene encoding Ole1p, an intrinsic membrane-bound -9 fatty acid desaturase required for the synthesis of oleic acid in yeast cells [32].

**Table 4:** Pterostilbene-responsive genes involved in lipid metabolism

**Accession**	**Gene**	**Ratio**	***p*-value**	**Gene Description**
a. Lipid biosynthesis/metabolism process				
YBR004C	*GPI18*	3.1	1.28 × 10^-4^	Functional ortholog of human PIG-V
YGL126W	*SCS3*	2.4	1.06 × 10^-3^	Required for inositol prototrophy
YDR173C	*ARG82*	2.3	2.53 × 10^-4^	Inositol polyphosphate multikinase (IPMK)
YGR036C	*CAX4*	2.3	0.02	Dolichyl pyrophosphate (Dol-P-P) phosphatase
YIL124W	*AYR1*	-2.7	6.79 × 10^-4^	1-acyl dihydroxyacetone phosphate reductase
YJL153C	*INO1*	10.0	6.73 × 10^-4^	L-myo-inositol-1-phosphate synthase
YJL196C	*ELO1*	-2.8	1.18 × 10^-5^	Elongase
YKL051W	*SFK1*	4.0	4.36 × 10^-4^	Suppressor of PI Four Kinase
YMR165C	*PAH1*	2.2	1.15 × 10^-3^	Mg^2+^-dependent phosphatidate (PA) phosphatase
YMR207C	*HFA1*	2.0	3.22 × 10^-4^	Mitochondrial acetyl-coenzyme A carboxylase
YNL169C	*PSD1*	-2.1	1.93 × 10^-3^	Phosphatidylserine decarboxylase
YNL231C	*PDR16*	6.1	3.54 × 10^-4^	by the multiple drug resistance regulator Pdr1p
YNR043W	*MVD1*	-2.2	3.55 × 10^-4^	Mevalonate pyrophosphate decarboxylase
YPR183W	*DPM1*	-2.3	2.88 × 10^-4^	Dolichol phosphate mannose synthase
YKR009C	*FOX2*	3.1	7.82 × 10^-4^	Multifunctional beta-oxidation protein
YLR284C	*ECI1*	2.1	8.91 × 10^-3^	Peroxisomal Δ3, Δ2-enoyl-CoA isomerase
YML042W	*CAT2*	2.4	2.21 × 10^-4^	Carnitine O-acetyltransferase
YMR246W	*FAA4*	2.1	1.25 × 10^-4^	Long chain fatty acyl-CoA synthetase
YNR019W	*ARE2*	4.1	1.28 × 10^-4^	Acyl-CoA:sterol acyltransferase
YOR100C	*CRC1*	2.4	0.01	Carnitine transporter
YKR031C	*SPO14*	2.1	1.36 × 10^-3^	Phospholipase D
YMR008C	*PLB1*	3.9	3.37 × 10^-4^	Phospholipase B (lypophospholipase)
b. Sphingolipid metabolism				
YBR265W	*TSC10*	-2.4	5.46 × 10^-4^	3-ketosphinganine reductase
YCR034W	*FEN1*	-3.0	3.49 × 10^-4^	Fatty acid elongase
YDR062W	*LCB2*	-2.1	5.13 × 10^-4^	Component of serine palmitoyltransferase
YDR072C	*IPT1*	6.5	2.64 × 10^-6^	Inositolphosphotransferase 1
YLR260W	*LCB5*	3.7	8.66 × 10^-4^	Minor sphingoid long-chain base kinase
YOR171C	*LCB4*	2.6	8 × 10^-5^	Sphingoid long-chain base kinase
YPL057C	*SUR1*	3.5	4.67 × 10^-3^	Probable catalytic subunit of a mannosylinositol phosphorylceramide (MIPC) synthase
YPL087W	*YDC1*	2.2	2.93 × 10^-3^	Alkaline dihydroceramidase
c. Genes involved in the regulation of lipid biosynthesis and metabolism				
YAL051W	*OAF1*	2.0	1.1 × 10^-3^	Oleate-activated transcription factor
YDR096W	*GIS1*	4.0	2.12 × 10^-3^	Transcriptional factor
YDR213W	*UPC2*	8.8	6.78 × 10^-5^	Sterol regulatory element binding protein
YOR237W	*HES1*	3.1	4.32 × 10^-3^	Protein implicated in the regulation of ergosterol biosynthesis;
YIR033W	*MGA2*	2.0	1.53 × 10^-3^	ER membrane protein involved in regulation of OLE1 transcription
YKL020C	*SPT23*	2.1	7.73 × 10^-4^	ER membrane protein involved in regulation of OLE1 transcription
YOR049C	*RSB1*	131.8	2.23 × 10^-4^	Suppressor of sphingoid long chain base (LCB) sensitivity of an LCB-lyase mutation
YOL108C	*INO4*	2.1	5.1 × 10^-3^	Transcription factor required for derepression of inositol-choline-regulated genes

Pterostilbene exposure also resulted in a significant up-regulation of genes associated with the ontology term "response to drug" (*z*-score of 5.05 – Table 3; genes listed in Table 5). Three of these genes encode transcription factors, including *PDR3 *involved in the regulation of the pleiotropic drug resistance (PDR) response in yeast [33], *YAP1*, encoding a basic leucine zipper transcription factor required for oxidative stress tolerance [34], and *YRR1*, encoding a Zn2-Cys6 zinc-finger transcription factor that activates genes involved in multidrug resistance [35]. Notably, the significantly induced genes in this category were transporters, especially the ABC transporters whose function is known to be involved in multidrug resistance against a broad range of antifungal agents [36]. It should be also noted that the *AZR1 *gene, which encodes a plasma membrane protein required for adaptation to acetic acid and resistance to azole drugs such as ketoconazole and fluconazole in yeast [37], was induced by more than 73-fold (Table 5).

**Table 5:** Pterostilbene-responsive genes associated with "response to drug" gene ontology category

**Accession**	**Gene**	**Ratio**	***p*-value**	**Molecular Function**
a. Multidrug resistance ABC transporters				
YOR153W	*PDR5*	6.1	6.01 × 10^-5^	Multidrug resistance ABC transporter
YOR328W	*PDR10*	7.1	1.01 × 10^-4^	ABC transporter highly similar to Pdr5p
YGR281W	*YOR1*	10.3	1.01 × 10^-4^	Multidrug resistance ABC transporter
YDR011W	*SNQ2*	8.2	9.71 × 10^-6^	Multidrug resistance ABC transporter
b. Multidrug resistance MFS transporters				
YNL065W	*AQR1*	2.2	7.19 × 10^-3^	MFS-multidrug resistance transporter
YGR224W	*AZR1*	73.4	2.16 × 10^-4^	MFS plasma membrane transporter
c. Multidrug resistance transcription factors				
YBL005W	*PDR3*	9.5	4.22 × 10^-4^	Transcriptional activator of the PDR network
YML007W	*YAP1*	3.1	1.66 × 10^-5^	Transcription factor required for oxidative stress
YOR162C	*YRR1*	2.5	9.44 × 10^-5^	Transcription factor involved in MDR
d. Other drug-responsive genes				
YOR266W	*PNT1*	2.0	0.01	Mitochondrial protein involved in export of proteins
YNL231C	*PDR16*	6.1	3.54 × 10^-4^	Pdr17p homolog controlled by Pdr1p
YGR197C	*SNG1*	4.1	3.7 × 10^-4^	Protein involved in nitrosoguanidine resistance
YOR018W	*ROD1*	2.8	8.67 × 10^-3^	Membrane protein, resistance to *o*-dinitrobenzene

Including the transcription factors discussed above involved in the regulation of lipid metabolism and pleiotropic drug resistance, in total more than 30 transcription factors were up-regulated in response to pterostilbene (see Additional file 3: List of pterostilbene-responding genes in the "cell wall," "transcription factor activity," and "mitochondrion" categories). Of particular interest are genes encoding transcription factors involved in mitochondrial function (*RTG1 *and *RTG2*), cell wall-related functions (*RLM1*), and methionine metabolism (*MET4 *and *MET31*), considering that these functional categories were over-represented in the gene ontology analysis (Table 3). Thus, the genes present in these functional categories include transcription factors as well as their cognate targets, suggesting the modulation of these processes, at least in part, via altered levels of these specific transcription factors in response to pterostilbene exposure.

**Figure 2:**
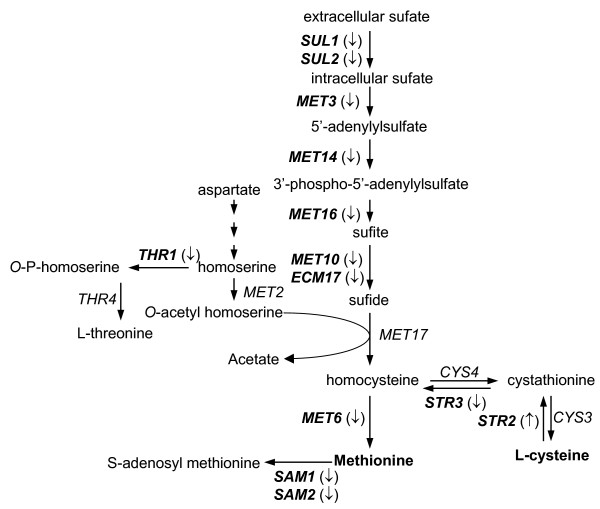
Summary of gene expression responses to pterostilbene in the sulfur metabolism pathway. Methionine biosynthetic pathway and corresponding genes are based on Thorsen et al. [66] and the *Saccharomyces *Genome Database [25]. Genes shown in boldface in the pathway are those affected by pterostilbene. The up- and down-regulated genes were indicated by arrows, (↑) and (↓).

In the Cellular Component-based ontology analysis, genes required for mitochondrial and cell wall-related functions were also over-represented (Table 3). The cell wall category had a *z*-score of 8.22 (Table 3), and most of the genes assigned within this category were down-regulated by pterostilbene treatment (Table 3). The majority of these genes are involved in mating responses [e.g., *FIG1*, *FIG2*, *SAG1*, *AGA1*, *MF(ALPHA)1*, and *MF(ALPHA)2*, see Additional file 3: *op. cit*.], which is also in agreement with the over-representation of the gene ontology term "reproduction" in the Biological Process category (*z*-score of 3.01, Table 3). Additional down-regulated cell wall-related genes included chitin synthase 2(*CHS2*), cell wall mannoproteins (*TIP1, CWP1*) and a major exo-1,3-beta-glucanase of the cell wall (*EXG1*) (see Additional file 3: *op. cit*.). These genes are responsible for the biosynthesis of three major constituents (chitin, mannoproteins and glucan) found in yeast cell walls [38]. The gene *BAG7 *(Rho GTPase activating protein), however, was induced by ~137-fold in pterostilbene treated cells (see Additional file 3: *op. cit*.). It has been reported that Bag7p plays a role in the control of cell wall synthesis and is involved in regulating key components of the cell wall stress-response pathway [39].

With respect to mitochondrial genes, it is interesting to note that more than 100 genes involved in diverse mitochondrial functions were up-regulated by pterostilbene treatment (Table 3, and Additional file 3: *op. cit*.). These genes play important roles in respiration, electron transport, mitochondrial protein targeting, and mitochondrial protein synthesis (see Additional file 3: *op. cit*.).

Of particular interest was the finding that a group of genes involved in sulfur metabolism was significantly over-represented, generating a *z*-score of 10.5 for the category "sulfur amino acid metabolism," (the highest score obtained in the present study). Within this category, a subset of genes assigned to the ontology term "methionine metabolism" (*z*-score of 11.78) were down-regulated upon exposure to pterostilbene (Table 3). Of the 21 genes assigned to this ontology term on the array, eleven were differentially regulated. As shown in Figure 2 and Table 6, all responsive genes involved in sulfur-containing amino acid biosynthesis were down-regulated, with the exception of *MET4 *(transcriptional activator) and *MET31 *(transcriptional regulator), which were up-regulated (Table 6).

**Table 6:** Pterostilbene-responsive genes involved in sulfur metabolism and/or assimilation

**Accession**	**Gene**	**Ratio**	***p*-value**	**Gene Description**
a. Methionine metabolism				
YJR137C	*ECM17*	-2.5	2.48 × 10^-5^	Sulfite reductase beta subunit
YKR069W	*MET1*	-3.1	3.88 × 10^-4^	AdoMet-dependent uroporphyrinogen III transmethylase
YJR010W	*MET3*	-3.8	3.5 × 10^-4^	ATP sulfurylase
YER091C	*MET6*	-2.4	8.83 × 10^-5^	Cobalamin-independent methionine synthase
YFR030W	*MET10*	-2.1	1.5 × 10^-3^	Sulfite reductase alpha subunit
YGL125W	*MET13*	-3.6	1.46 × 10^-4^	Methylenetetrahydrofolate reductase
YKL001C	*MET14*	-3.1	1.12 × 10^-3^	Adenylylsulfate kinase
YPR167C	*MET16*	-2.0	7.56 × 10^-4^	3'-phosphoadenylylsulfate reductase
YOL064C	*MET22*	-2.5	1.68 × 10^-4^	Bisphosphate-3'-nucleotidase,
YLL062C	*MHT1*	-3.4	6.69 × 10^-4^	S-methylmethionine-homocysteine methyltransferase
YLR180W	*SAM1*	-2.1	6.39 × 10^-5^	S-adenosylmethionine synthetase
b. Sulfur and aspartate family amino acid metabolic processes (in addition to a)				
YNL103W	*MET4*	3.0	4.01 × 10^-4^	Leucine zipper transcriptional activator
YPL038W	*MET31*	2.2	2.45 × 10^-3^	Transcriptional regulator highly homologous to Met32p
YJR130C	*STR2*	2.3	2.97 × 10^-3^	Cystathionine gamma-synthase
YGL184C	*STR3*	-3.6	4.1 × 10^-3^	Cystathionine beta-lyase
YJR060W	*CBF1*	-3.0	1.11 × 10^-3^	Basic helix-loop-helix protein
YHR025W	*THR1*	-2.2	2.05 × 10^-4^	Homoserine kinase
c. All other genes affected under the term "sulfur metabolism"				
YLL060C	*GTT2*	2.5	8.23 × 10^-4^	Glutathione transferase
YEL017W	*GTT3*	-2.2	6.72 × 10^-4^	Protein with a possible role in glutathione metabolism
YGR286C	*BIO2*	-3.0	9.79 × 10^-4^	Biotin synthase
YJL212C	*OPT1*	-2.5	1.8 × 10^-4^	Peptide transporter/glutathione transporter
YLL057C	*JLP1*	13.5	6.93 × 10^-4^	Fe(II)-dependent sulfonate/α-ketoglutarate dioxygenase
YLR043C	*TRX1*	-2.1	6.95 × 10^-5^	Cytoplasmic thioredoxin
d. Sulfur uptake				
YBR294W	*SUL1*	-5.53	4.64 × 10^-5^	Sulfate uptake (transporter)
YLR092W	*SUL2*	-2.75	1.89 × 10^-4^	High affinity sulfate permease

### Validation of microarray data by quantitative real-time RT-PCR

**Figure 3:**
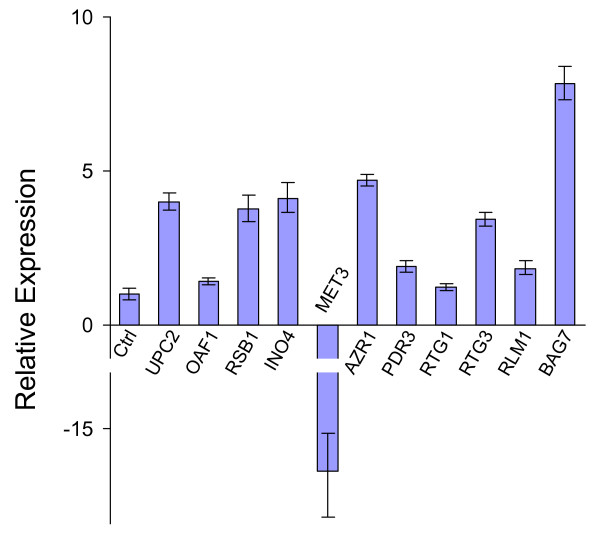
Quantitative real-time RT-PCR analysis of genes identified as differentially expressed by microarray experiments. Assays were performed in triplicate as described previously [18]. Data were normalized to an internal control (18S rRNA) and the ΔΔC_T _method was used to obtain the relative expression level for each gene. Data are shown as mean ± standard deviation (SD). "Ctrl" represents samples treated with solvent (0.25% DMSO) alone.

For further verification of the transcript profiling results, selected genes were analyzed by quantitative real time RT-PCR with cDNAs prepared from the identical RNA samples used for microarray target preparations. For these analyses, eleven genes associated with gene ontology categories discussed above were selected, including genes involved in lipid metabolism (*UPC2*, *OAF1*, *RSB1 *and *INO4*), sulfur metabolism (*MET3*), response to drug (*AZR1 *and *PDR3*), mitochondrial functions (*RTG1 *and *RTG3*) and cell wall-related functions (*RLM1 *and *BAG7*). The results (Figure 3) are in agreement with the microarray experiments, with ten genes showing up-regulation and one gene (*MET3*) showing down-regulation in response to pterostilbene treatment. A few discrepancies in fold-change values between real-time RT-PCR and microarray analyses could be attributed to technical differences between the two methods. For example, the genes *RSB1*, *AZR1*, and *BAG7 *showed a higher level of induction in the microarray data compared to the real-time RT-PCR data. This discrepancy could be attributed to the fact that the Affymetrix software assigned an "absent" call to these genes in the untreated sample – thus the signal values assigned were artifactually low in the untreated sample compared to the treated sample, making the fold-change values very high [18]. Other discrepancies could be attributed to the greater dynamic range of real-time RT-PCR compared to microarray analysis.

### Sensitivity of yeast mutants to pterostilbene

**Figure 4:**
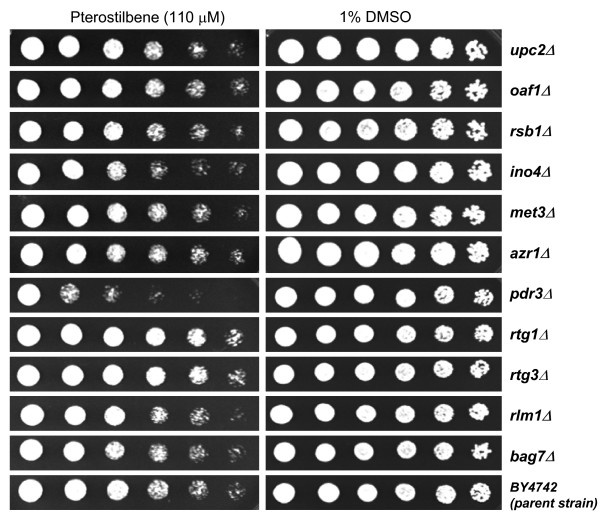
Growth of selected yeast mutant strains in the presence of pterostilbene. A parent strain BY4742 and 11 mutants (*upc2Δ*, *oaf1Δ*, *rsb1Δ*, *ino4Δ*, *met3Δ*, *azr1Δ*, *pdr3Δ, rtg1Δ*, *rtg3Δ*, *rlm1Δ*, and *bag7Δ*) were analyzed. Serial dilutions of overnight cultures were spotted onto solid YPD (pH 7.0, MOPS buffered) agar plates containing either 1% DMSO or 110 μM pterostilbene, and the plates were photographed after incubation for 2 days at 30°C.

In order to further investigate the relationship between the gene expression responses to pterostilbene and their phenotypic consequences in yeast mutants, we analyzed a number of yeast mutants with deletions in selected genes from each of the categories discussed above for their sensitivity to pterostilbene. The same genes selected for real-time RT-PCR analysis were also chosen for this study, and these included genes involved in lipid metabolism (*UPC2*, *OAF1*, *RSB1 *and *INO4*), sulfur metabolism (*MET3*), response to drug (*AZR1 *and *PDR3*), mitochondrial functions (*RTG1 *and *RTG3*) and cell wall-related functions (*RLM1 *and *BAG7*). As shown in Figure 4, the loss of Pdr3p (mutant *pdr3Δ*) resulted in increased sensitivity to pterostilbene, while the loss of Upc2p, Oaf1p, Bsr1p, Ino4, Met3p, Azr1p, Rlm1p or Bag7p resulted in neither increased nor decreased sensitivity to pterostilbene under the experimental conditions used. Surprisingly, the loss of Rtg1p or Rtg3p resulted in slightly increased resistance to the compound.

## Discussion

Stilbenes, such as resveratrol and pterostilbene, represent biologically active secondary metabolites of plants known to have diverse pharmacological properties, which include cancer-chemopreventive activity. They are also considered to be phytoalexins due to their potent antifungal activities [14-16]. To investigate the molecular pathways affected by pterostilbene in eukaryotes, we conducted DNA microarray analysis using the model yeast *S. cerevisiae*, and identified large-scale transcriptome alterations associated with pterostilbene exposure in this organism. The responsive genes are associated with a diversity of biological, molecular and cellular processes, suggesting that pterostilbene exerts wide-ranging effects on the physiological and biochemical processes of eukaryotic cells (Tables 2 and 3).

Pterostilbene affected the expression of genes involved in sulfur metabolism and/or sulfur assimilation, associated with the highest *z*-score in this study (Tables 3 and 6). Within this category, six genes (*ECM17*, *MET3, MET14, MET16, MET10, MET6*) encoding enzymes involved in methionine biosynthesis were repressed (Figure 2, Table 6). Methionine metabolism plays an important role in many aspects of cellular physiology. Its derivative S-adenosyl methionine (SAM) serves as a methyl donor in many biosynthetic processes [40]. Metabolism of sulfur-containing amino acids like methionine has been linked to cell cycle progression, and perturbations of these processes lead to diverse cellular anomalies [41,42]. The down-regulation of genes involved in methionine biosynthesis by pterostilbene may cause cellular stress by not only decreasing methionine levels, but also by compromising the supply of donor methyl groups required for methylation reactions in various biosynthetic pathways. Our results suggest that one of the molecular effects of pterostilbene might involve the disruption of methionine biosynthesis, an observation that has not been previously reported for this compound.

The observed down-regulation of genes involved in methionine metabolism in response to pterostilbene treatment (Figure 2, Table 6) is perhaps surprising given that several genes in this pathway are actually up-regulated during conditions of oxidative stress and amino acid starvation [43,44]. However, a transcriptional profiling study conducted in yeast cells exposed to the herbicide sulfometuron methyl (SM), which inhibits branched-chain amino acid biosynthesis, indicated that exposure to SM resulted in the down-regulation of several genes involved in methionine metabolism, including *MET3*, *MET6*, *MET14*, *SAM1*, and *SAM2 *[45]. One mechanism proposed for this down-regulation was the potentially reduced levels of ATP in SM-treated cells, given that methionine biosynthesis and the production of SAM are ATP-requiring processes. Interestingly, in the present work pterostilbene treatment altered the expression of more than 100 mitochondrial genes (Additional file 3: *op. cit*.), suggesting large-scale perturbations in mitochondrial function which would eventually lead to ATP deficiency. A second potential mechanism emerged from queries using *MET1*, *MET3*, *MET6*, *MET10*, *MET13*, *MET14*, and *MET16 *against the Serial Pattern of Expression Levels Locator (SPELL) database [46], which revealed that all of the corresponding transcripts are down-regulated in response to osmotic stress [47]. Given the significant effects pterostilbene exposure is likely to have on lipid metabolism (Table 4), it is possible that membrane integrity could be compromised leading to an osmotic imbalance in yeast cells. Consistent with this notion, genes involved in osmotic stress response regulation such as *GRE1*, *GRE2*, *SSK1*, *PPZ1*, and *STE11 *were induced in pterostilbene-treated cells (Additional file 3: *op. cit*.).

Of further significance, the present results show that pterostilbene up-regulated OAF1, which encodes a transcription factor that regulates the expression of genes involved in the beta-oxidation of fatty acids in peroxisomes in yeast cells [28]. In addition, genes encoding enzymes required for fatty acid β-oxidation were also up-regulated by pterostilbene. It has been previously shown that pterostilbene lowers lipid/lipoprotein levels in hypercholestrolemic hamsters through activation of the peroxisome proliferator-activated receptor α (PPARα) [10]. PPARα is involved in fatty acid and lipid metabolism, through the activation of genes involved in fatty acid β-oxidation in the liver, heart, kidney and skeletal muscles [10,48,49]. Thus, the up-regulation of genes involved in fatty acid beta-oxidation by pterostilbene in the present report is consistent with previous observations of its effects on mammalian cells. In addition, pterostilbene also up-regulated several genes involved in sterol, phospholipid and sphingolipid metabolism, including genes involved in the regulation of lipid metabolism. Taken together, these results suggest that lipid metabolism is likely to be an important molecular pathway that is affected by pterostilbene.

Transcript levels of a number of genes involved in the pleiotropic/multiple drug resistance response were also found to increase dramatically following pterostilbene exposure (Table 5). These genes include ABC transporters, multidrug resistance transcription factors, and other drug-responsive genes. The plasma membrane-associated efflux pumps Pdr5p and Snq2p are under the genetic control of the transcription factors Pdr1p and Pdr3p [50]. In yeast cells exposed to pterostilbene, the transcripts of *PDR3*, *PDR5 *and *SNQ2 *increased 9.5-, 6.1- and 8.2-fold, respectively (Table 5). In addition, a yeast mutant with a deletion in the *PDR3 *gene showed strong hypersensitivity to pterostilbene, confirming the importance of this gene in conferring resistance to this compound (Figure 4). Notably, the ABC transporter *YOR1*, involved in mediating the export of many different organic anions including oligomycin, was also up-regulated 10.3-fold in pterostilbene-treated cells. Moreover, the expression of a plasma membrane transporter, encoded by *AZR1*, which is involved in the resistance to azole drugs, dramatically increased by 73.4-fold following exposure. Taken together, the strong up-regulation of this set of transporters suggests that drug efflux is one of the major mechanisms involved in the cellular detoxification of pterostilbene in yeast cells.

As mentioned above, an additional outcome from the present work is the observation of the up-regulation of a large number of genes involved in diverse mitochondrial functions, including mitochondrial respiration, mitochondrial protein synthesis and mitochondrial protein targeting (associated with a *z*-score of 6.1 – Tables 3, and Additional file 3: *op. cit*.). Furthermore, *RTG1 *and *RTG3*, genes encoding transcription factors that play an important role in regulating the communication between the mitochondria and the nucleus in yeast cells, were up-regulated by pterostilbene. Mitochondria are vital for energy production, and their disruption in humans has been implicated in aging, diabetes, heart disease, and various neurodegenerative disorders [51]. Recently, one of the mechanisms by which pterostilbene induces apoptosis in human gastric carcinoma cells was found to involve the activation of the caspase cascade via the mitochondrial pathway [52]. Thus, the induction of mitochondrial genes by pterostilbene in the present study is apparently consistent with its effects on this organelle in human cancer cell lines.

Of the eleven mutants tested carrying deletions in selected genes from pterostilbene-responsive pathways, only the *pdr3Δ *mutant showed hypersensitivity to pterostilbene (Figure 4). Lack of correlation between transcriptional profiling and deletion mutant analyses has been reported in the literature by investigators using both selected individual mutants as well as whole-genome mutant populations [53-57]. Such discrepancies could be attributable to factors such as post-transcriptional regulation of target genes [56], functional redundancy [53], as well as the specific nature of the inhibitor tested (e.g., genes required for survival in the presence of the inhibitor could be distinct from those which are transcriptionally responsive; [55]). Nevertheless, the hypersensitivity to pterostilbene exhibited by the *pdr3Δ *mutant in the present study, coupled with elevated levels of *PDR3 *as well as other multidrug resistance-related transcripts (Figure 4, Table 5), confirms the importance of efflux-mediated detoxification pathways in the cellular response to this compound. Further mechanistic insights could potentially be gained by extending these analyses to whole-genome mutant collections of *S. cerevisiae *generated by The *Saccharomyces *Genome Deletion Project [57], which have been successfully used by others for the identification of molecular targets of various clinically relevant drugs, as well as for the investigation of interactions between the cellular pathways affected [58-61]. Such studies, coupled with transcriptional profiling experiments, could provide further corroborative evidence indicating the relevance of specific genes to the mechanism of action of pterostilbene.

The present study provides an overview of the major transcriptional responses to pterostilbene using the model eukaryote *S. cerevisiae*. While transcriptional profiling was employed as the principal tool to investigate the molecular effects of pterostilbene, an examination of the response of the yeast proteome would further refine our understanding of pterostilbene-induced gene expression changes, given that transcript and protein levels do not correlate in all cases [62-65]. Additionally, the transcriptional profiling results presented in the current work rely on a single drug concentration (IC_50_) and time point (one doubling), thus it is likely that many of the transcriptional responses observed represent indirect consequences of drug exposure. An expansion of the current data set to include multiple time points and drug concentrations could therefore prove invaluable in distinguishing between the primary and secondary effects of pterostilbene, such as mechanism-related vs. adaptation-related cellular responses. Nevertheless, the present work yields important clues and provides a foundation for further studies directed towards elucidating the precise mechanism of action of pterostilbene.

## Conclusion

We have identified the molecular pathways affected by pterostilbene, and our results show that pterostilbene affects the expression of a diverse group of genes in yeast cells. Using Gene Ontology-based analysis, the most significant effects were observed in genes involved in "methionine metabolism," "response to drug," "transcription factor activity," and "mitochondrion" functions. Additional analyses indicated that many genes involved in lipid metabolism were also affected. The observed response of lipid metabolism genes is in agreement with the known hypolipidemic properties of pterostilbene mediated through the activation of PPARα. The induction of a large number of mitochondrial genes by pterostilbene is consistent with its previously-demonstrated role in apoptosis in human cancer cells. Our data also show that pterostilbene has a significant effect on methionine metabolism, perhaps resulting in the depletion of methionine by the inhibition of methionine biosynthesis. The effect of pterostilbene on methionine metabolism has not been previously observed and merits further investigation.

## Competing interests

The author(s) declare that they have no competing interests.

## Authors' contributions

ZP conceived and coordinated the study, performed the data analysis and drafted the manuscript. AKA participated in the design of the study, contributed to the data analysis, and helped to draft the manuscript. TX carried out the yeast mutant sensitivity assays and RT-PCR primer design. SRB performed the real-time quantitative RT-PCR experiments. AMR synthesized the compound for this study. QF performed the IC_50 _determination and the DNA microarray experiments. SOD provided intellectual input and guidance, and assisted with manuscript preparation. All authors read and approved the final manuscript.

## Pre-publication history

The pre-publication history for this paper can be accessed here:



## Supplementary Material

Additional file 1 Correlation between expression ratios obtained from quantitative real-time RT-PCR and microarray experiments. Click here for file

Additional file 2 List of all significant pterostilbene-responding genes. Click here for file

Additional file 3 List of pterostilbene-responding genes in the "cell wall," "transcription factor activity," and "mitochondrion" categories. Click here for file
