# Supplementary material for: Identification of molecular pathways affected by pterostilbene, a natural dimethylether analog of resveratrol
Source: BMC Med Genomics. 2008 Mar 20;1:7. doi: 10.1186/1755-8794-1-7 (PMC2330146; doi:10.1186/1755-8794-1-7)
Supplement: Additional file 1 — Correlation between expression ratios obtained from quantitative real-time RT-PCR and microarray experiments. [file 1755-8794-1-7-S1.pdf]

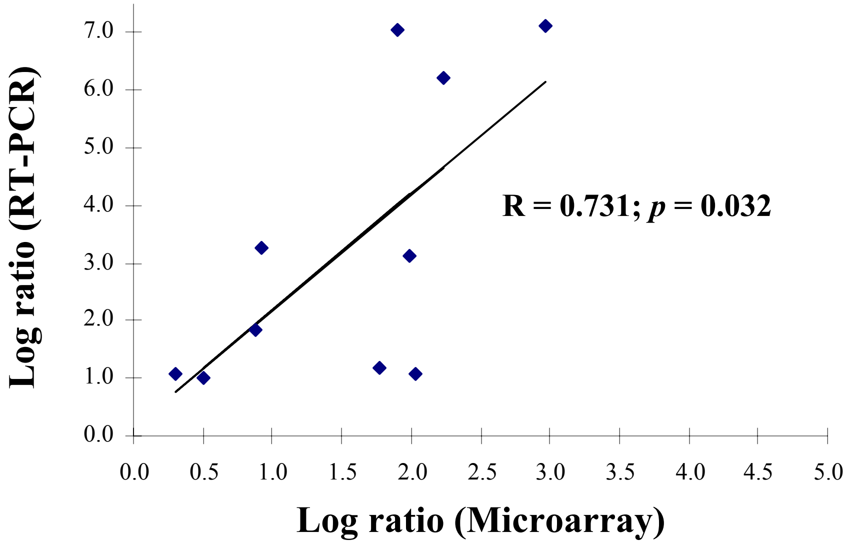

Additional File 3. Correlation between expression ratios obtained from quantitative real-time RT-PCR and microarray experiments. Linear regression analysis was performed for selected pterostilbene-induced genes using $\log_2$-transformed RT-PCR relative expression values ($2^{-\Delta\Delta C_T}$; *y*-axis) and mean relative expression values obtained by microarray analysis (*x*-axis). *p*-value indicates the significance of the correlation as determined by an F-test, R= correlation coefficient generated for the theoretical line of best fit (shown as black line in above plot).
